# Unusual Presentation of Childhood Leukemia With Vaginal Bleeding: A Case Report

**DOI:** 10.1155/crh/9949125

**Published:** 2025-05-20

**Authors:** Şule Çalışkan Kamış

**Affiliations:** Adana Faculty of Medicine, Adana City Training and Research Hospital, Pediatric Hematology and Oncology Clinic, University of Health Sciences, Adana, Turkey

**Keywords:** B-cell acute lymphoblastic leukemia, case report, thrombocytopenia, vaginal bleeding

## Abstract

**Introduction:** This case report describes a 17-year-old female patient who initially presented with vaginal bleeding, weight loss, and nonspecific symptoms, which led to the diagnosis of B-cell acute lymphoblastic leukemia (B-ALL). This unusual presentation highlights the importance of considering hematological malignancies in patients with atypical symptoms.

**Case Presentation:** The patient, married for 2 weeks, experienced vaginal bleeding following her first sexual intercourse, which did not resolve spontaneously. She also reported a 6 kg weight loss over the past 3-4 months, hair loss, and a history of dysmenorrhea and an ovarian cyst detected 2 years prior. Laboratory investigations revealed leukocytosis (WBC: 16,500/μL), anemia (Hb: 11.4 g/dL), and thrombocytopenia (Plt: 44,000/μL). Bone marrow aspiration (BMA) and flow cytometry confirmed the diagnosis of B-ALL, revealing a high percentage of atypical lymphoid cells.

**Discussion:** This case underscores the rarity of diagnosing hematological malignancies in patients with vaginal bleeding. The patient's symptoms, including weight loss and thrombocytopenia, should have prompted a more comprehensive evaluation. Early recognition of B-ALL is crucial as prompt treatment significantly improves outcomes. The patient was started on prednisolone and alkalinized fluids, and her condition was closely monitored.

**Conclusion:** Vaginal bleeding in young patients should not be dismissed as a minor issue, especially when accompanied by other systemic symptoms like weight loss and thrombocytopenia. Early diagnosis of B-ALL in such cases can lead to better management and prognosis.

## 1. Introduction

This case report presents a 17-year-old female patient who was initially referred with vaginal bleeding and was subsequently diagnosed with B-cell acute lymphoblastic leukemia (B-ALL). The patient's clinical and laboratory findings, along with the treatment process, are discussed in this report.

## 2. Case Report

### 2.1. Chief Complaints

A 17-year-old girl was referred from an external center to the pediatric emergency department due to vaginal bleeding and weight loss. The bleeding began one day after her first sexual intercourse and did not resolve spontaneously. She underwent surgical repair for a suspected vaginal tear at the external center and was given blood product support.

She had no prior episodes of bleeding. Additional complaints included appetite loss, a 6 kg weight loss over the past 3-4 months, and hair loss. Two years earlier, she had presented to a gynecology clinic with dysmenorrhea and irregular menstruation, during which a 4.5 cm ovarian cyst was detected.

### 2.2. Physical Examination

The patient was alert, pale, and in stable condition, with no signs of hemodynamic compromise. She was conscious and cooperative. No hepatosplenomegaly was noted upon physical examination.

### 2.3. Laboratory Findings

The complete blood count revealed leukocytosis (WBC: 16,500/μL), normocytic anemia (Hb: 11.4 g/dL and MCV: 82 fL), and thrombocytopenia (Plt: 44,000/μL). Vitamin B12 and ferritin levels were 397 pg/mL and 128 ng/mL, respectively. Coagulation parameters were within normal limits. A peripheral smear showed the presence of atypical reactive lymphocytes.

Viral serologies including Epstein–Barr virus (EBV) viral capsid antigen (VCA) IgM, cytomegalovirus (CMV) IgM, parvovirus B19 IgM, and hepatitis B surface antigen (HBsAg) were all negative. Hemoglobin electrophoresis demonstrated HbA at 97.4% and HbA2 at 2.6%. LDH was 242 U/L, and uric acid was 4.42 mg/dL. Von Willebrand factor antigen and ristocetin cofactor activity were 89.5% and 86.34%, respectively, while Factor VIII activity was 74.7%. A summary of the laboratory results is presented in Table 1, including reference ranges.

### 2.4. Bone Marrow Aspiration (BMA) and Flow Cytometry

**Figure 1:**
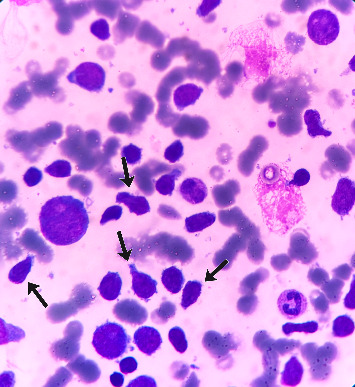
Bone marrow smear showing L2-type blasts with mirror image-like morphological features.

BMA revealed L2-type blasts with mirror image-like morphological features (Figure 1), consistent with acute lymphoblastic leukemia (ALL). The blasts were characterized by larger cell size, irregular nuclear contours, and prominent nucleoli, which further supported the diagnosis.

**Figure 2:**
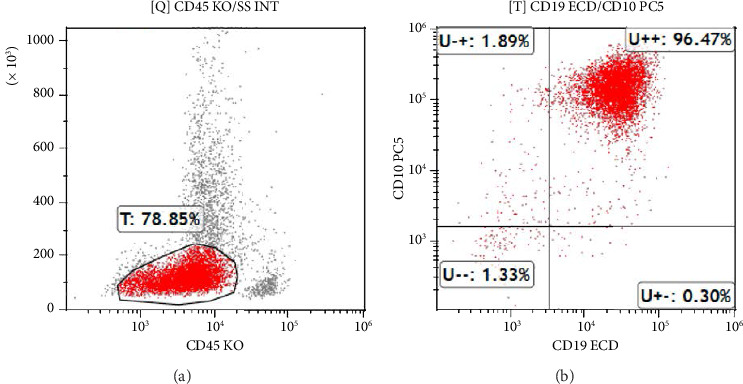
Flow cytometry analysis of bone marrow sample. Flow cytometry analysis demonstrating CD45 and CD19/CD10 expression patterns. (a) CD45 gating, highlighting 78.85% of the blast population. (b) CD19 and CD10 coexpression, with 96.47% of the gated cells positive for both markers, supporting the diagnosis of B-cell acute lymphoblastic leukemia (B-ALL).

Flow cytometry analysis identified 78.85% of an atypical CD45dim lymphoid-derived cell population. This atypical population expressed TdT, cCD79a, CD10, CD19, CD22, CD58, CD38, and CD34, confirming the diagnosis of B-ALL (Figure 2). The immunophenotypic profile was consistent with a precursor B-cell origin, in accordance with the WHO Classification of Haematolymphoid Tumors, 5th Edition (2022).

### 2.5. Echocardiography and Electrocardiogram (ECG)

Echocardiography findings were normal. The ECG showed respiratory arrhythmia without pathological findings. No contraindications for chemotherapy were noted.

### 2.6. Treatment and Monitoring

The patient was diagnosed with B-ALL and admitted to the pediatric hematology-oncology unit. The Berlin–Frankfurt–Münster (BFM)-ALL 2009 chemotherapy protocol was initiated. This protocol includes multiple treatment phases—induction, consolidation, reinduction, and maintenance—and involves a combination of corticosteroids (e.g., prednisolone), vincristine, L-asparaginase, anthracyclines (e.g., daunorubicin), methotrexate, and 6-mercaptopurine. Treatment is risk adapted based on early response, minimal residual disease (MRD), and cytogenetic findings. On Day 15, the MRD result was negative, indicating a good early treatment response. The patient is currently being closely monitored for further chemotherapy cycles and potential long-term complications.

### 2.7. Cytogenetic and Molecular Findings

Molecular testing was performed as part of the initial diagnostic workup. The Breakpoint Cluster Region–Abelson (BCR–ABL) p190 transcript was detected with a normalized copy number (NCN) of 280, indicating Philadelphia chromosome-positive (Ph+) B-ALL. In contrast, BCR–ABL p210 was negative. Fluorescence in situ hybridization (FISH) analysis revealed t(9; 22) (q34; q11) translocation in 63% of the analyzed cells, further supporting the diagnosis of Ph + B-ALL. Following the detection of the BCR-ABL p190 transcript, the treatment plan was modified to include dasatinib, a tyrosine kinase inhibitor (TKI), in addition to the chemotherapy regimen.

Given the high-risk classification associated with this molecular profile, hematopoietic stem cell transplantation (HSCT) was considered early in the treatment course. Human leukocyte antigen (HLA) typing was performed, and a fully matched sibling donor was identified among the patient's adult siblings.

## 3. Discussion

Vaginal bleeding in adolescents is a common symptom with a wide range of differential diagnoses, including hormonal imbalances, trauma, or gynecological conditions such as ovarian cysts or fibroids [1]. However, in this case, vaginal bleeding, along with anemia and thrombocytopenia, prompted a more thorough investigation, revealing an underlying hematologic malignancy B-ALL. While hematological malignancies in pediatric patients are often characterized by symptoms such as fatigue, fever, bone pain, and pallor, the association between vaginal bleeding and leukemia is rarely discussed in the literature [2, 3].

Ph+ B-ALL, defined by the presence of the BCR–ABL fusion gene, typically presents with bone marrow failure symptoms, particularly bleeding tendencies due to thrombocytopenia. However, case reports directly linking BCR–ABL positivity to specific bleeding presentations are scarce. For instance, El Shafie et al. (2022) reported a 52-year-old woman who presented with fatigue, bone pain, and abnormal vaginal bleeding, later diagnosed with Ph + T-ALL, in which vaginal bleeding was the initial manifestation [4]. Similarly, Mroczkowska et al. (2022) described an 11-year-old boy with severe, refractory epistaxis, subsequently diagnosed with Ph + B-ALL carrying a rare e8a2 BCR–ABL1 fusion transcript [5]. These reports demonstrate that, although rare, BCR–ABL-positive ALL can initially present with bleeding symptoms. However, the limited number of such cases precludes establishing a definitive causal relationship between BCR–ABL positivity and bleeding risk.

In the present case, a 17-year-old girl experienced persistent vaginal bleeding following her first sexual intercourse, which ultimately led to the diagnosis of BCR–ABL-positive B-ALL. This case adds to the scarce literature emphasizing that Ph + ALL can present atypically with bleeding as the first clinical sign.

Acute lymphoblastic leukemia, particularly B-ALL, is the most common type of leukemia in children and adolescents [6, 7]. It often presents with symptoms due to bone marrow failure, including anemia, thrombocytopenia, and neutropenia, resulting in clinical manifestations such as fatigue, weakness, increased susceptibility to infections, and easy bruising or bleeding [8, 9]. However, in this case, the patient's initial complaint of vaginal bleeding following sexual intercourse was unusual and led to a misdiagnosis of vaginal trauma. The patient's thrombocytopenia could explain the bleeding tendency; however, it was only after a detailed clinical and laboratory workup that the underlying leukemia was identified.

The patient's laboratory findings, including leukocytosis, anemia, and thrombocytopenia, raised suspicion for a hematological malignancy, leading to further testing [10, 11]. The BMA and flow cytometry findings were crucial in confirming the diagnosis of B-ALL. The flow cytometry analysis identified a predominance of atypical lymphoid-derived cells expressing markers commonly associated with B-cell lineage, such as CD19, CD22, and CD10, along with a high percentage of terminal deoxynucleotidyl transferase (TdT)-positive cells, confirming the diagnosis of B-ALL. Cytogenetic analysis revealing the t(9;22) translocation further confirmed the diagnosis of Ph + B-ALL.

Notably, the presence of an ovarian cyst detected 2 years prior to the diagnosis, along with weight loss, hair loss, and persistent fatigue, could suggest an ongoing underlying process that might have predisposed the patient to leukemia. Although the ovarian cyst was likely a benign finding, its coexistence with other systemic symptoms may have delayed the correct diagnosis. Weight loss and hair loss may be attributable to both the underlying malignancy and the effects of bone marrow failure, which are commonly observed in pediatric leukemia patients.

It is important to note that BCR–ABL fusion, by activating tyrosine kinase pathways, may theoretically influence vascular integrity and coagulation, although current evidence predominantly associates bleeding with marrow failure rather than direct molecular effects [12]. Nonetheless, rare cases, as highlighted in the literature, suggest that atypical bleeding presentations in Ph + ALL should raise clinical suspicion, especially when unexplained by trauma alone.

Adolescents with leukemia may present with nonspecific symptoms that mimic other common conditions, often delaying diagnosis. This case underscores the importance of a broad differential diagnosis and comprehensive clinical evaluation in adolescents presenting with unexplained bleeding or unusual symptoms.

Although vaginal bleeding is an uncommon initial symptom in pediatric hematologic malignancies, clinicians should still consider leukemia in the differential diagnosis—particularly when accompanied by hematologic abnormalities. Prompt recognition and intervention are essential for improving patient outcomes. This case also illustrates the critical role of multidisciplinary collaboration—pediatric hematologists, gynecologists, and emergency care teams were instrumental in ensuring timely diagnosis and appropriate management.

In conclusion, while vaginal bleeding is not commonly associated with hematologic malignancies, this case demonstrates the importance of recognizing B-ALL in adolescents presenting with atypical symptoms. The clinical findings, including vaginal bleeding, anemia, and thrombocytopenia, along with laboratory investigations and bone marrow analysis, provided essential clues for the timely diagnosis and initiation of treatment. Recognizing atypical presentations and integrating detailed clinical, laboratory, and molecular findings are vital for optimal outcomes in pediatric leukemia patients.

## 4. Conclusion

This case highlights the importance of considering B-ALL in adolescents presenting with unexplained vaginal bleeding and cytopenias. Early diagnosis and multidisciplinary management are essential to improve outcomes, and this report adds to the limited literature on atypical bleeding presentations in Ph + B-ALL.

## Figures and Tables

**Table 1:** Initial laboratory findings of the patient.

Parameter	Result	Unit	Reference range
WBC	16.5	× 10^3^/μL	4.0–11.0 × 10^3^/μL
Hb	11.4	g/dL	12–16 g/dL
MCV	82	fL	80–96 fL
Plt	44	× 10^3^/μL	150–450 × 10^3^/μL
Vitamin B12	397	pg/mL	180–914 pg/mL
Ferritin	128	ng/mL	10–291 ng/mL
LDH	242	U/L	125–220 U/L
Uric acid	4.42	mg/dL	2.6–6.0 mg/dL
vWF antigen	89.5	%	50%–150%
Ristocetin cofactor	86.34	%	50%–150%
Factor VIII activity	74.7	%	50%–150%
HbA	97.4	%	95%–98%
HbA2	2.6	%	1.5%–3.5%

*Note:* Hb, hemoglobin; HbA, hemoglobin A; HbA2, hemoglobin A2; LDH, lactate dehydrogenase; Plt, platelet.

Abbreviations: MCV, mean corpuscular volume; vWF, von willebrand factor; WBC, white blood cell.

## Data Availability

The data supporting the findings of this article are available from the corresponding author upon reasonable request.

## Nomenclature

B-ALL: B-cell acute lymphoblastic leukemia
Ph+: Philadelphia chromosome-positive
BCR–ABL: Breakpoint Cluster Region–Abelson
TKI: Tyrosine kinase inhibitor
HSCT: Hematopoietic stem cell transplantation
HLA: Human leukocyte antigen
MRD: Minimal residual disease
BMA: Bone marrow aspiration
TdT: Terminal deoxynucleotidyl transferase
CD: Cluster of differentiation
ECG: Electrocardiogram
BFM: Berlin–Frankfurt–Münster
FISH: Fluorescence in situ hybridization
vWF: von Willebrand factor
WBC: White blood cell
Hb: Hemoglobin
MCV: Mean corpuscular volume
Plt: Platelet
LDH: Lactate dehydrogenase
HbA: Hemoglobin A
HbA2: Hemoglobin A2
